# The Efficacy of Traditional Chinese Medical Exercise for Parkinson’s Disease: A Systematic Review and Meta-analysis

**DOI:** 10.1371/journal.pone.0122469

**Published:** 2015-04-01

**Authors:** Yan Yang, Wei Qing Qiu, Yan Lei Hao, Zhan Yun Lv, Shu Ji Jiao, Jun Feng Teng

**Affiliations:** 1 Department of Neurology, Affiliated Hospital of Jining Medical University, Jining, Shandong, China; 2 Department of Neurology, Laizhou People’s Hospital of Shangdong, Laizhou, Shandong, China; 3 Binzhou Medical University Hospital, Binzhou, Shandong, China; 4 Department of Neurology, The First People’s Hospital of Hebi, Hebi, Henan, China; Duke University, UNITED STATES

## Abstract

**Background and Objective:**

Several studies assessed the efficacy of traditional Chinese medical exercise in the management of Parkinson’s disease (PD), but its role remained controversial. Therefore, the purpose of this systematic review is to evaluate the evidence on the effect of traditional Chinese medical exercise for PD.

**Methods:**

Seven English and Chinese electronic databases, up to October 2014, were searched to identify relevant studies. The PEDro scale was employed to assess the methodological quality of eligible studies. Meta-analysis was performed by RevMan 5.1 software.

**Results:**

Fifteen trials were included in the review. Tai Chi and Qigong were used as assisting pharmacological treatments of PD in the previous studies. Tai Chi plus medication showed greater improvements in motor function (standardized mean difference, SMD, -0.57; 95% confidence intervals, CI, -1.11 to -0.04), Berg balance scale (BBS, SMD, -1.22; 95% CI -1.65 to -0.80), and time up and go test (SMD, -1.06; 95% CI -1.44 to -0.68). Compared with other therapy plus medication, Tai Chi plus medication also showed greater gains in motor function (SMD, -0.78; 95% CI -1.46 to -0.10), BBS (SMD, -0.99; 95% CI -1.44 to -0.54), and functional reach test (SMD, -0.77; 95% CI -1.51 to -0.03). However, Tai Chi plus medication did not showed better improvements in gait or quality of life. There was not sufficient evidence to support or refute the effect of Qigong plus medication for PD.

**Conclusions:**

In the previous studies, Tai Chi and Qigong were used as assisting pharmacological treatments of PD. The current systematic review showed positive evidence of Tai Chi plus medication for PD of mild-to-moderate severity. So Tai Chi plus medication should be recommended for PD management, especially in improving motor function and balance. Qigong plus medication also showed potential gains in the management of PD. However, more high quality studies with long follow-up are warrant to confirm the current findings.

## Introduction

Parkinson’s disease (PD) is a common neurodegenerative disorder with insidious onset. There is an estimation of at least 4 million people diagnosed as PD worldwide [1]. In China, PD is 1.70% in prevalence rate among people aged more than 65 years old [2]. Although the specific causes of PD are under investigation, incidence increases with age, especially after 50 years old [3]. The landmark symptoms of PD are resting tremor, bradykinesis, rigidity and decreased postural reflexes. These impairments lead to a decline in functional status as gait disturbance and balance decrements so that people with PD cannot cope with their daily tasks well [4–5]. It is reported that this decrease in functional status worsens as the disease progresses and often results in loss of independence and a decline in quality of life [6].

Although the precise reasons of the decrease in balance, gait and quality of life are still unknown, exercise is a preventive strategy that has demonstrated efficacy in PD [7,8]. Traditional Chinese medical exercise, including Qigong, Tai Chi, Wuqinxi, etc., combines body movements with mental focus. Tai Chi and Qigong, as representative traditional Chinese medical exercises, incorporate body movement, breath and mind training to maintain health and remove disease symptoms. Tai Chi, with slow body positions and dance-like movements that flow from one to the next continuously, promotes posture, flexibility, relaxation, well-being and mental concentration [9]. The difference between Qigong and Tai Chi is that Tai Chi is a martial art with movements practiced quickly which can provide self-defense and are externally focused. Meanwhile, Qigong cannot and it is internally focused [10].

In the last decade, Tai Chi and Qigong have been studied in the management of PD [11–15], but there were conflicting results. Li et al. reported significant improvements in balance, functional capacity and falls after Tai Chi exercise [14]. In contrast, Amano et al. concluded that Tai Chi was not effective in improving parkinsonian disability [15]. And previous reviews did not show consistent and strong evidence of Tai Chi for PD [16–19]. What’s more, there was no comprehensive systematic review of traditional Chinese medical exercise for PD.

Therefore, the aim of this systematic review is to summarize and evaluate the evidence on the efficacy of traditional Chinese medical exercise for PD. To our knowledge, this is the first comprehensive systematic review summarizing the effect of traditional Chinese medical exercise for PD patients, focusing on motor function, gait and quality of life. Based on our findings, recommendations for future research are offered.

## Methods

### Search Strategy

The relevant studies were retrieved from the following online databases up to October 2014: PubMed, EMBASE, OVID-MEDLINE, Cochrane Library, China Knowledge Resource Integrated Database, Weipu Database for Chinese Technical Periodicals and Wan Fang Data. The following keywords were used: Parkinson, Parkinson’s disease, Parkinsonism, traditional Chinese medical exercise, Tai Chi, Qigong, Wuqinxi, Baduanjin and Yijinjing. WHO International Clinical Trials Registry Platform, ProQuest Dissertations and Chinese Dissertation Full-text Database were also searched to identify unpublished studies. And we contacted experts in relevant field. The literature search was performed independently by two authors (S Jiao and ZY Lv), and disagreements were resolved by discussion.

### Study Selection

Two authors (Y Yang and WQ Qiu) independently identified and selected the studies based on standardized manner. The studies that met the following criteria were included: (1) study design: randomized controlled trials (RCTs) and non-randomized controlled trials (non-RCTs); (2) the target population was diagnosed as PD in any stage; (3) traditional Chinese medical exercise was practiced alone or combined with stable medication, such as Madopar, compared to placebo, no intervention and any other therapies with or without stable medication; (4) the primary outcomes were motor function assessed by Unified Parkinson’s Disease Rating Scale III (UPDRS III), health related quality of life assessed by Parkinson’s Disease Questionnaire-39 (PDQ-39) or Activities of Daily Living (ADL), balance assessed by Berg Balance Scale (BBS), Functional Reach Test (FRT) or Time Up and Go Test (TUG) and gait assessed by gait velocity, stride/step length, or 6-Minute Walking Test (6-MWT); (5) the studies contain available data for the meta-analysis; (6) the paper was available in either English or Chinese. Any disagreement was settled by discussion or by consulting a third author (J Teng).

### Data Extraction

Two authors (Y Yang and WQ Qiu) independently performed data extraction from the eligible studies. The following information was extracted: (1) study source and study design; (2) patients characteristics: sample size, age, gender and disease stage; (3) details of the interventions: type, duration, dose and frequency; (4) main outcomes and (5) length of follow-up. For the crossover study, the first phase of the study was adopted for the sake of prohibiting carryover effects. The primary author was contacted by e-mails when the relevant data was not reported. Any disagreement was settled by discussion or by consulting a third author (YL Hao).

### Quality Assessment

Two authors (Y Yang and S Jiao) independently assessed the methodological quality of eligible studies using PEDro scale. The PEDro score has a fair-to-good reliability for the physiotherapy studies in systematic reviews [20,21]. And higher scores represent a better quality. The necessary information in eligible studies was supplemented by contacting the corresponding authors. There was no disagreement between the authors regarding PEDro scores.

### Data Synthesis and Analysis

Meta-analysis was conducted with Cochrane Collaboration software (Review Manager Version 5.1). D-value of the pre and post treatment was used as the change of curative effect. As for three or four-armed studies, the similar control groups have be merged with computational formula provided by the Cochrane handbook to create a single pair-wise comparison. For continuous data, standardized mean difference (SMD) and 95% confidence intervals (CI) of random-effects model were calculated for all eligible trials. The *I* ^*2*^ statistic, a quantitative measure of inconsistency across studies, was employed in assessing heterogeneity. Heterogeneity was regarded high if the *I* ^*2*^ is greater than 75%. Detailed subgroup analyses were performed to compare Tai Chi/Qigong plus medication with medication alone or other therapy plus medication. Publication bias was assessed using funnel plot if the group included more than 10 studies.

## Results

### Study Selection

**Fig 1:**
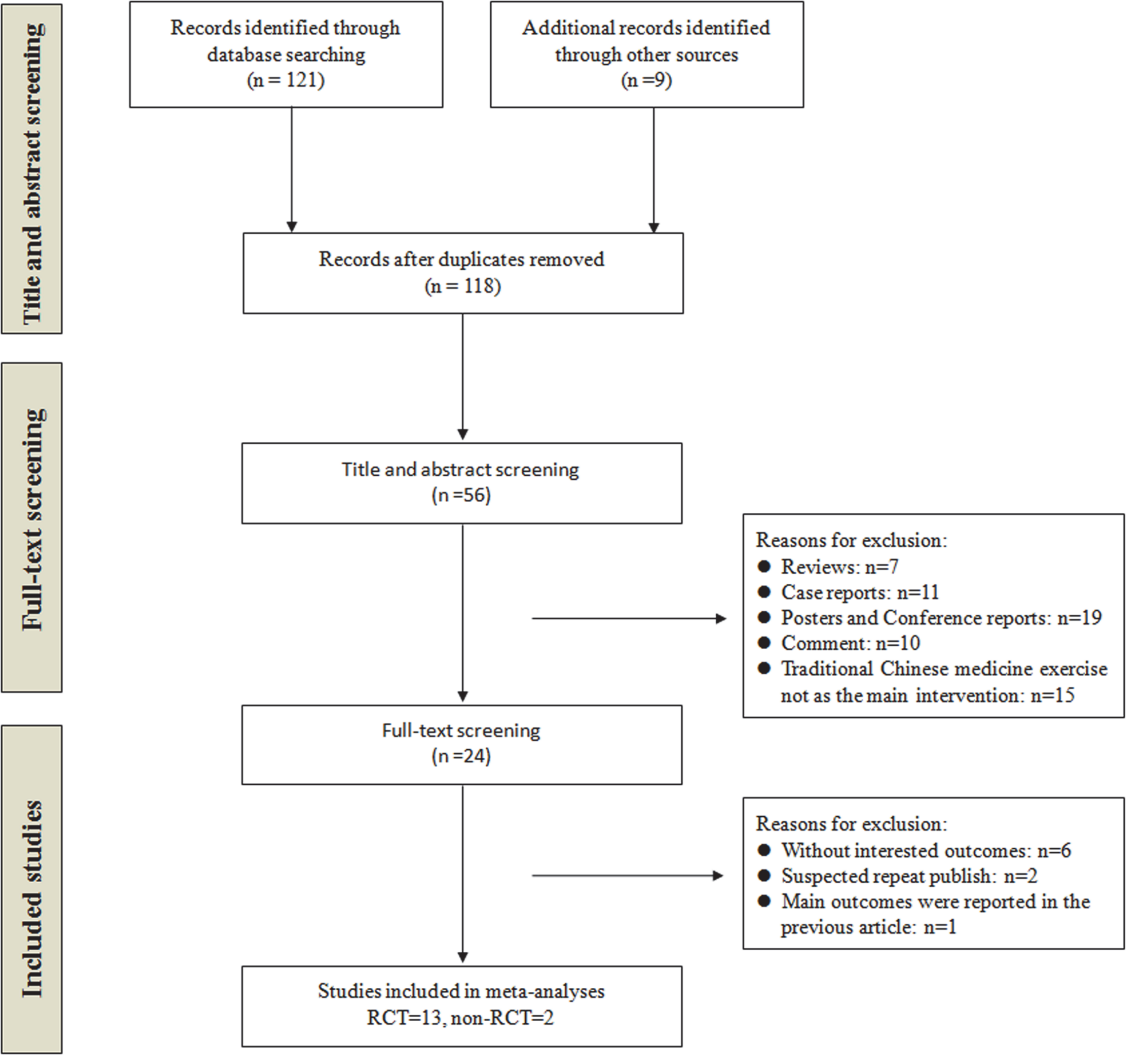
Flow diagram of study selection. RCT: randomized controlled trial.

A total of 118 records were identified after removing duplicates. During the preliminary screening of the titles and abstracts, 62 studies were eliminated. After full-texts screening, 13 RCTs [11,13–15,21–25,27–31] and 2 non-RCTs [12,26] were included in our review. 9 studies were published in English [13–15,22–24,27,28,31] and 6 in Chinese [11,12,25,26,29,30]. The studies were excluded due to without interested outcomes (n = 6), suspected repeat publication (n = 2) and repeated report of main outcomes (n = 1). The detailed process of search and identification was shown in Fig 1.

### Study Characteristics

A total of 799 participants with the mean age of 64.57 ± 4.88 years were included. The patients in most studies were diagnosed as PD of mild-to-moderate severity. The patients in 10 studies [13,15,22–28,31] were diagnosed as Hoehn and Yahr stage I to III and patients in 2 studies [14,30] were Hoehn and Yahr stage I to IV. The other studies [11,12,28] did not report the Hoehn and Yahr stage of eligible patients, but the patients can finish Qigong or Tai Chi exercise independently. Qigong [11–13,29] or Tai Chi [14,15,22–28,30–31] was employed as assisting pharmacological therapies in the included studies. The control interventions included medication [11,12,15,22,23,27,28,30,31], stretching/resistance training plus medication [14], dancing plus medication [23], walking plus medication [25,26] and other exercises plus medication [13,24,27,29]. The intervention time ranged from 4 weeks to 50 weeks. The details of study characteristics were summarized in Table 1.

**Table 1:** Characteristics of the included studies.

Study source	Design	Sample size	Mean age (years)	Gender (M/F)	Disease stage (Hoehn and Yahr stage)	Follow-up (weeks)	Duration (weeks)	Main outcome	Experimental group intervention	Control group intervention
Yu 1998, China [11]	RCT	83	58±11;71±10	38/14;29/2	NR	—	50	Webster scale	Qigong plus medication (30min/700sessions)	Medication
Gu 2002, China [12]	Non RCT	51	57±9;51±12	23/10;17/7	NR	36	12	Webster scale	Qigong plus medication (30min/168sessions)	Medication
Burini 2006, Italy [13]	RCT	26	65.7±7;62.7±4	5/8;4/9	2–3	—	7	UPDRS, 6-MWT, PDQ-39	Qigong plus medication(45min/20sessions)	Aerobic exercise plus medication(45min/20sessions)
Hackney 2008, US [22]	RCT	26	64.9±8.3;61.7±10.1	12/2; 9/3	2±0.46;2±0.3	—	13	UPDRS III, BBS, Gait, TUG, 6-MWT	Yang-style Tai Chi plus medication(60min/20sessions)	Medication
Hackney 2009, US [23]	RCT	61	66.8±2.4;68.2±1.4;64.9±2.3;66.5±2.8	11/6;11/3;11/2;12/5	2.0±0.2;2.1±0.1;2.0±0.1;2.2±0.2	—	13	PDQ-39	Yang-style Tai Chi plus medication(60min/20sessions)	1) Tango plus medication;2) Waltz/Foxtrot plus medication(60min/20sessions);3) Medication
Gladfelter 2011, US [24]	RCT	17	72±8.52	12/5	2.4±0.87	—	12	BBS, FRT, TUG, PDQ-39	Yang-style Tai Chi plus medication(60min/12sessions)	Physical exercise plus medication
Li 2011, China [25]	RCT	47	68.28±6.62; 67.13±6.73	11/13;11/12	2.5–3	—	8	UPDRS III, BBS, PDQ-39	Tai Chi plus medication(30-45min/80sessions)	Walking plus medication(40min/80sessions)
Zhu 2011, China [26]	RCT	38	63.35±8.72; 64.83±9.29	11/9;12/8	1–2	—	4	UPDRS III, BBS	Tai Chi plus medication(30-45min/40sessions)	Walking plus medication(40min/40sessions)
Li 2012, US [14]	RCT	195	68±9; 69±8; 69±9	45/20; 38/27;39/26	1–4	12	24	UPDRS III, Gait, FRT, TUG	Tai Chi plus medication(60min/48sessions)	1) Stretching plus medication;2) Resistance training plus medication(60min/48sessions)
Amano 2013, US [15]	RCT	45	64±13; (66±11);68±7; 66±7	7/5(7/8);7/2;7/2	2.3±0.4(2.4±0.6);2.2±0.4;2.4±0.4	—	16	UPDRS III, Gait	1) Yang-style Tai Chi plus medication(60min/32-48sessions);2) Qigong plus medication(60min/32sessions)	Medication
Cheon 2013, Korea [27]	Non-RCT	23	62.3±6.5;65.6±7.9;64.9±7.2	0/23	2–3	—	8	UPDRS, ADL	Sun-style Tai Chi plus medication(50-65min/24sessions)	1) Medication2) Exercise plus medication(60min/24sessions)
Choi 2013, Korea [28]	RCT	20	60.81±7.6;65.54±6.8	NR	1.6±0.6;1.8±0.3	—	12	UPDRS, TUG,6-MWT, OLS, ADL	Tai Chi plus medication(60min/36sessions)	Medication
Cheng 2014, China [29]	RCT	66	57±9;51±12	23/10;17/16	NR	—	12	UPDRS, 6-MWT	Qigong plus medication (60min/24sessions)	Routine exercise plus medication (60min/24sessions)
Gao 2014, China [30]	RCT	80	69.54±7.32;68.28±8.53	23/14;27/12	1–4	24	12	UPDRS III, BBS, TUG	Yang-style Tai Chi plus medication(60min/36sessions)	Medication
Nocera 2014, US [31]	RCT	21	66±11;65±7	7/8;4/2	2–3	—	16	PDQ-39	Yang-style Tai Chi plus medication(60min/48sessions)	Medication

RCT = randomized controlled trial; NR = no reported; Non-RCT = non-randomized controlled trial; 6-MWT = 6-minute walking test; UPDRS = unified Parkinson’s disease rating scale; BBS = berg balance scale; TUG = timed up and go; PDQ-39 = Parkinson’s disease questionnaire-39; FRT = functional reach test; ADL = activities of daily living; OLS = one-leg standing time.

### Methodological Quality

The methodological quality of the included studies was presented in Table 2. The total scores on the PEDro scale ranged from 3 to 8 points. Randomized allocation was employed in most studies (87%) [11,13–15,22–26,28–31], but only 4 trials were considered as allocation concealment due to lack of detailed descriptions [13,15,25,30]. None of the studies blinded the therapists or subjects, but independent assessors, who were unaware of the allocation, were employed in most studies (80%) [13–15,22–26,28–31]. 6 studies definitely showed a high expulsion rate over 15% [13,22–25,27]. And 4 studies used the intention-to-treat analysis [11,12,14,15]. The eligible studies showed good methodological quality in the remaining items of PEDro scale. Funnel plot analysis was not performed because none of the groups included more than 10 trials.

**Table 2:** PEDro scale of quality for included trials.

Study	Eligibility criteria	Random allocation	Concealed allocation	Similar atbaseline	Subjects blinded	Therapists blinded	Assessors blinded	<15%dropouts	Intention-to-treat analysis	Between-group comparisons	Point measures and variability data	Total
Yu 1998 [11]	1	1	0	1	0	0	0	1	1	1	1	6
Gu 2002 [12]	1	0	0	1	0	0	0	1	1	1	1	5
Burini 2006 [13]	1	1	1	1	0	0	1	0	0	1	1	6
Hackney 2008 [22]	1	1	0	1	0	0	1	0	0	1	1	5
Hackney 2009 [23]	1	1	0	1	0	0	1	0	0	1	1	5
Gladfelter 2011 [24]	1	1	0	1	0	0	1	0	0	1	1	5
Li 2011 [25]	1	1	1	1	0	0	1	0	0	1	1	6
Zhu 2011 [26]	1	1	0	1	0	0	1	1	0	1	1	6
Li 2012 [14]	1	1	0	1	0	0	1	1	1	1	1	7
Amano 2013 [15]	1	1	1	1	0	0	1	1	1	1	1	8
Cheon 2013 [27]	1	0	0	1	0	0	0	0	0	1	1	3
Choi 2013 [28]	1	1	0	1	0	0	1	1	0	1	1	6
Cheng 2014 [29]	1	1	0	1	0	0	1	1	0	1	1	6
Gao 2014 [30]	1	1	1	1	0	0	1	1	0	1	1	7
Nocera 2014 [31]	1	1	0	1	0	0	1	1	0	1	1	6

Criteria (2–11) were used to calculate the total PEDro score. Each criterion was scored as either 1 or 0 according to whether the criteria was met or not, respectively.

### The Effect of Tai Chi for PD

#### Motor function

**Fig 2:**
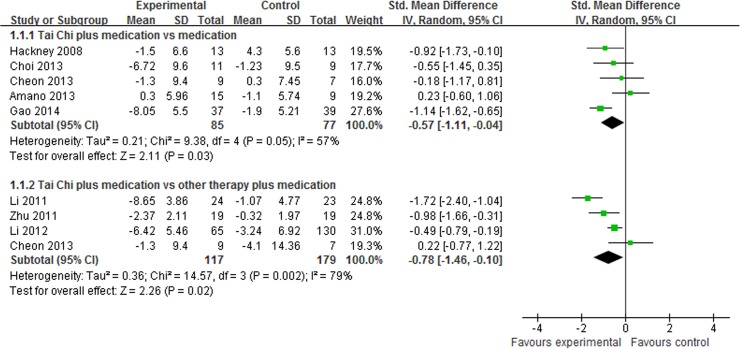
The effect of Tai Chi plus medication in motor function.

UPDRS III was reported in 8 studies [14,15,22,25–28,30], and subgroup analysis was performed. Most of them reported that Tai Chi plus medication showed beneficial effect in UPDRS III. The aggregated result also indicated that Tai Chi plus medication showed greater improvements in UPDRS III than medication alone (SMD, -0.57; 95% CI -1.11 to -0.04; p = 0.03, Fig 2) [15,22,27,28,30] and other therapy plus medication (SMD, -0.78; 95% CI -1.46 to -0.10; p = 0.02, Fig 2) [14,25–27].

#### Balance

**Fig 3:**
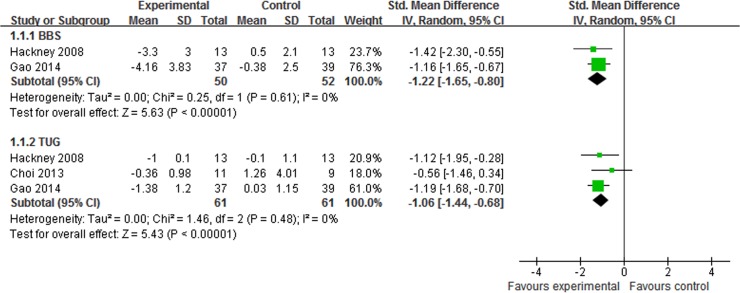
The effect of Tai Chi plus medication in balance compared with medication alone. BBS = berg balance scale; FRT = functional reach test;TUG = timed up and go.

**Fig 4:**
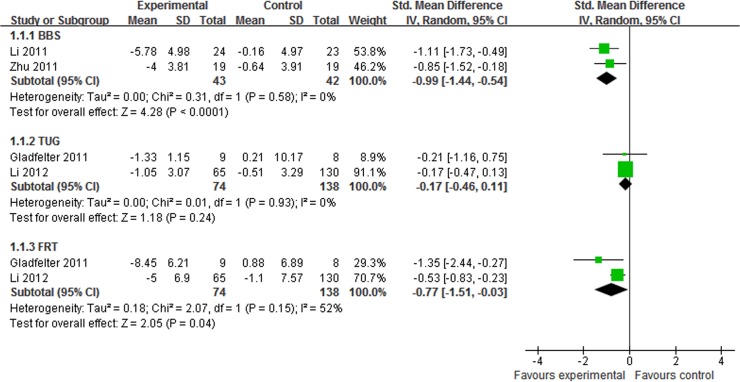
The effect of Tai Chi plus medication in balance compared with other therapy plus medication. BBS = berg balance scale; FRT = functional reach test; TUG = timed up and go.

7 studies assessed the effect of Tai Chi plus medication in improving balance in patients with PD [14,22,24–26,28,30]. The aggregated result indicated that Tai Chi plus medication showed greater improvements on BBS (SMD, -1.22; 95% CI -1.65 to -0.80; p<0.00001, Fig 3) [22,30] and TUG (SMD, -1.06; 95% CI -1.44 to -0.68; p<0.00001, Fig 3) [22,28,30] than medication alone. Compared with other therapy plus medication, Tai Chi plus medication also showed greater improvements on BBS (SMD, -0.99; 95% CI -1.44 to -0.54; p<0.0001, Fig 4) [25,26] and FRT (SMD, -0.77; 95% CI -1.51 to -0.03; p = 0.04, Fig 4) [14,24], but not on TUG (SMD, -0.17; 95% CI -0.46 to 0.11; p = 0.24, Fig 4) [14,24]

#### Gait

**Fig 5:**
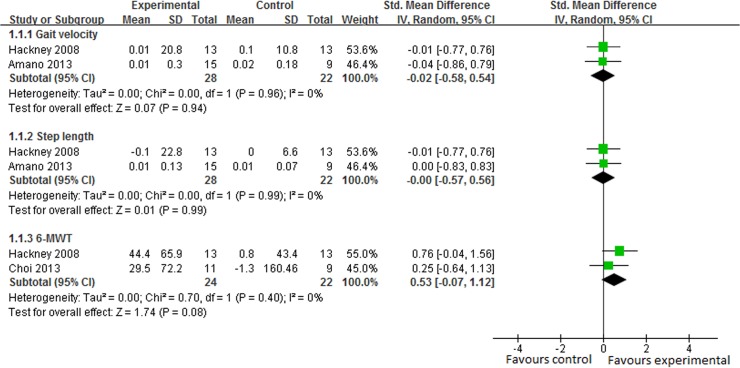
The effect of Tai Chi plus medication in gait velocity, step length and 6-minute walking test (6-MWT) compared with medication alone.

In 5 studies, gait function was assessed by gait velocity and step length [14,15,22] and gait endurance was assessed by 6-MWT [22,28]. The subgroup analysis suggested that there was no significant difference between Tai Chi plus medication and medication alone in gait velocity (SMD, -0.02 95% CI -0.58 to 0.54; p = 0.94, Fig 5) [15,22], step length (SMD, -0.00 95% CI -0.57 to 0.56; p = 0.99, Fig 5) [15,22] or 6-MWT (SMD, 0.53 95% CI -0.07 to 1.12; p = 0.08, Fig 5) [22,28]. However, one study reported that Tai Chi plus medication group performed better in gait velocity and step length than stretching plus medication group, and outperformed the resistance-training plus medication group in step length [14].

#### Quality of life

**Fig 6:**
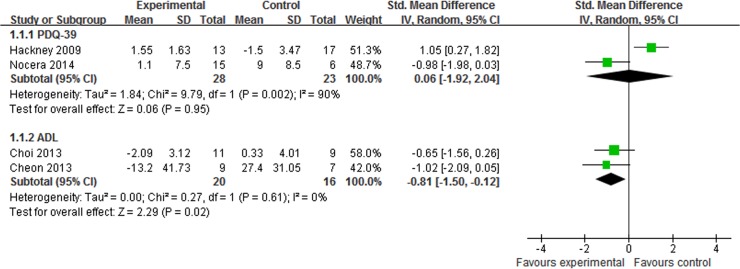
The effect of Tai Chi plus medication in quality of life compared with medication alone. PDQ-39 = Parkinson’s disease questionnaire-39; ADL = activities of daily living.

**Fig 7:**
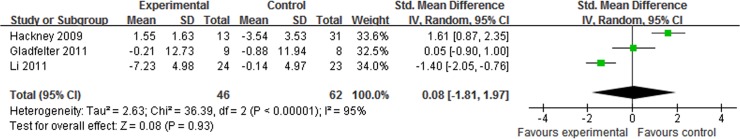
The effect of Tai Chi plus medication on Parkinson’s disease questionnaire-39 compared with other therapy plus medication.

The quality of life was assessed in 6 trials [23–25,27,28,31], and the subgroup analysis was performed. Tai Chi plus medication showed greater improvements than medication alone on ADL (SMD, -0.81 95% CI -1.50 to -0.12; p = 0.02, Fig 6) [27,28]. On PDQ-39, however, the aggregated result indicated that there was no significant difference between Tai Chi plus medication and medication alone (SMD, 0.06 95% CI -1.92 to 2.04; p = 0.95, Fig 6) [23,31] or other therapy plus medication (SMD, 0.08 95% CI -1.81 to 1.97; p = 0.93, Fig 7) [23–25].

### The Efficacy of Qigong for PD

#### Webster scale

**Fig 8:**
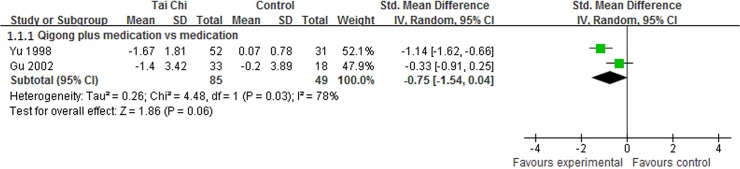
The effect of Qigong plus medication in Webster scale.

Webster scale is a comprehensive scale accessing clinical symptoms, quality of life and motor function in patients with PD. 2 studies reported that Qigong plus medication showed favorable improvements in Webster scale [11,12]. The meta-analysis indicated that Qigong plus medication demonstrated a small, but not statistically significant effect in Webster scale compared with medication alone (SMD, -0.75; 95% CI -1.54 to 0.04; p = 0.06, Fig 8) [11,12].

#### Motor function

**Fig 9:**
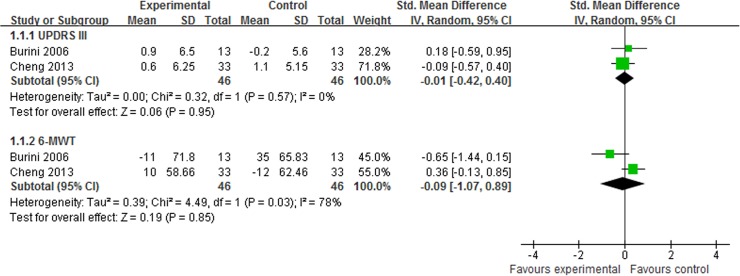
The effect of Qigong plus medication in Unified Parkinson’s Disease Rating Scale III (UPDRS III) and 6-minute walking test.

UPDRS III was reported in 3 trials [13,15,29]. One study reported that Qigong plus medication showed better effect than medication alone (UPDRS III mean changes: 3.4 versus 1.1) [15]. However, the meta-analysis showed that there was no significant difference between Qigong plus medication and other therapy plus medication (SMD, -0.01; 95% CI -0.42 to 0.40; p = 0.95, Fig 9) [13,29].

#### Gait

One study reported that Qigong plus medication did not show greater improvements than medication alone in gait velocity or step length [15]. Gait endurance was assessed by 6-MWT in 2 trials [13,29] and the meta-analysis was performed. The aggregated result showed that there was no significant difference between Qigong plus medication and other therapy plus medication (SMD, -0.09; 95% CI -1.07 to 0.89; p = 0.85, Fig 9) [13,29].

#### Quality of life

One study assessed the quality of life in patients with PD by PDQ-39, and reported that Qigong plus medication showed better effect than aerobic exercise plus medication (PDQ-39 mean changes: 2.8 versus -3.2) [13].

### Adverse Events

No serious adverse events were reported during the Tai Chi/Qigong training in eligible studies. Only one study reported that there were few back pain and ankle sprain [14].

## Discussion

This is the first comprehensive systematic review and meta-analysis to assess the effect of traditional Chinese medical exercise in the management of PD. Tai Chi and Qigong were used as assisting pharmacological treatments of PD in the previous studies. The positive finding was that Tai Chi plus medication showed greater gains than medication alone or other therapy plus medication in motor function and balance. However, there was not sufficient evidence on the efficacy of Tai Chi plus medication in improving gait or quality of life. Although some trials reported beneficial effect of Qigong plus medication for PD, the aggregated results did not support or refute it.

The positive finding of this systematic review should be available for patients with PD of mild-to-moderate severity due to most patients diagnosed as Hoehn and Yahr stage I to III [13,15,22–28,31]. All eligible patients can finish Tai Chi or Qigong exercise independently. And traditional Chinese medical exercise is based on the ability to stand and move independently. Therefore, these assisting pharmacological exercises should be recommended for PD patients of mild-to-moderate severity, especially in improving motor function and balance.

The last systematic reviews of Tai Chi for PD concur with our positive findings [17,18]. One supported that Tai Chi plus medication resulted in promising gains in mobility and balance for PD patients at an early stage [18]. However, there were serious limitations in this review. Firstly, two control interventions were considered as no intervention [15] and routine physical exercise [24] respectively, but stable medications were not changed during the study according to the author’s reply. Secondly, some subgroup analyses only included one trial. It was not valid because the meta-analysis should be performed based at least on two studies. What’s more, the similar control groups should be combined to create a single pair-wise comparison according to Cochrane handbook, but it was not performed in this review. The other review concluded that Tai Chi was a valid complementary and alternative therapy for PD, especially on motor function and balance [17]. However, Tai Chi as an assisting pharmacological treatment was not compared with medication alone or other therapy plus medication. In our review, detailed subgroup analyses were performed to compare Tai Chi plus medication with medication alone or other therapy plus medication.

Our results were different from some previous reviews [16,19]. Lee’s review concluded that there was no sufficient evidence to support Tai Chi in the management of PD [16], but it was only a qualitative review including 3 RCTs [32–34], 1 non-RCT [35], and 3 uncontrolled clinical trials [36–38] published between 1997 and 2007. And most of them were published by conference abstracts without detailed information [32–36]. Although the meta-analysis was performed in Toh’s systematic review [19], it only included 4 RCTs [14,15,22,23]. The main suspected reason of the difference was that numerous studies of Tai Chi for PD were published from 2008 to 2014 [14,15,22–28,30,31]. So current update provided stronger evidence of Tai Chi plus medication for PD.

Our result of Qigong for PD was supported by previous review [39]. The evidence was insufficient to support Qigong plus medication for PD due to limited number of studies. However, the beneficial finding of current review was that Qigong plus medication showed potential gains for PD. Two trials reported that Qigong plus medication showed better effect than medication alone in Webster scale [11,12], and one study reported that Qigong plus medication was superior to aerobic exercise plus medication in UPDRS III and PDQ-39 [13]. What’s more, it has been reported that Qigong has beneficial effects in improving physical performance, figure, quality of life, etc. [40–42]. Consequently, Qigong may be a valid assisting pharmacological treatment of PD. Further high quality RCTs are required to confirm current beneficial finding.

In previous studies, only Tai Chi and Qigong were focused, but other traditional Chinese medical exercises should also be investigated, such as Baduanjin and Wuqinxi. Li and his colleagues have reported beneficial effects of Baduanjin exercise in physical flexibility of healthy older [43]. Baduanjin has been recommended as a safe and feasible treatment option for patients with knee OA in disability, stiffness and quality of life [44]. Wuqinxi exercise may be a valid alternative treatment for low back pain in improving dysfunction [45], and for knee OA in balance function [46]. So Baduanjin and Wuqinxi exercise may be a valid assisting pharmacological treatment for PD.

Assuming that traditional Chinese medical exercise was beneficial for PD, some complex neurophysiological mechanisms may provide possible rationales [47–50]. Intensive exercise showed beneficial effects on neural plasticity, neuroprotection and preventing neural degeneration [47]. Especially, some animal studies have reported that intensive exercise may promote neurogenesis, dopamine synthesis and release in the striatum [51,52]. And such neural changes may affect behavioral recovery in individuals with PD [53,54]. In relevant studies, the intervention was generally considered as intensive exercise when involving 2–3 hours of exercise per week for 6–14 weeks (a total of 12–42 hours of treatment) [47]. In our systematic review, all eligible studies employed intensive traditional Chinese medical exercise (a total of 12–300 hours) for PD. And the intensive Tai Chi/Qigong also showed beneficial effects in improving motor function and balance. In addition, repetitive traditional Chinese medical exercise may also promote development of new motor programs which allow faster reactions responding to postural challenge [55]. And these new motor programs, which promoted behavioral recovery, may be due to making new synaptic connections.

There were some potential limitations in our systematic review: 1) there was the degree of uncertainty in locating relevant studies because of limited retrieving resources, language barrier, publication bias, etc. 2) there were few studies in some subgroup analyses because of strict eligibility criteria, which may bias the aggregated results. However, low eligibility criteria would conduct more doubtful results. 3) PEDro score was less than 6 in 5 studies. They were not considered as high quality, but they contributed valuable information to the evidence of Tai Chi/Qigong for PD. So they were included in our review. 4) synthetic results may be affected by different parameters (duration, frequency, dosage, etc.) of Tai Chi/Qigong exercise. 5) the follow-up effect of Tai Chi/Qigong for PD was not investigated in current studies. So further studies of Tai Chi/Qigong for PD should include long-term follow-up. 6) few adverse events were reported in included studies, but it was not concluded that Tai Chi/Qigong exercise was safe.

## Conclusions

In the previous studies, Tai Chi and Qigong were used as assisting pharmacological treatments of PD. The current systematic review showed positive evidence of Tai Chi plus medication for PD of mild-to-moderate severity. So Tai Chi plus medication should be recommended for PD management, especially in improving motor function and balance. Qigong plus medication also showed potential gains in the management of PD. However, more high quality studies with long follow-up are warrant to confirm the current findings. And relevant mechanism research of traditional Chinese medical exercise for PD is also required.

## Supporting Information

S1 PRISMA Checklist PRISMA Checklist. (DOC)
